# Mobility, exposure, and epidemiological timelines of COVID-19 infections in China outside Hubei province

**DOI:** 10.1038/s41597-021-00844-8

**Published:** 2021-02-05

**Authors:** Xiao Fan Liu, Xiao-Ke Xu, Ye Wu

**Affiliations:** 1 Web Mining Laboratory, Department of Media and Communication, City University of Hong Kong, Hong Kong Special Administrative Region, China; 2 College of Information and Communication Engineering, Dalian Minzu University, Dalian, 116600 China; 3 Computational Communication Research Center, Beijing Normal University, Zhuhai, 519087 China; 4 School of Journalism and Communication, Beijing Normal University, Beijing, 100875 China

**Keywords:** Epidemiology, Infectious diseases

## Abstract

The 2019 coronavirus disease (COVID-19) is pseudonymously linked to more than 100 million cases in the world as of January 2021. High-quality data are needed but lacking in the understanding of and fighting against COVID-19. We provide a complete and updating hand-coded line-list dataset containing detailed information of the cases in China and outside the epicenter in Hubei province. The data are extracted from public disclosures by local health authorities, starting from January 19. This dataset contains a very rich set of features for the characterization of COVID-19’s epidemiological properties, including individual cases’ demographic information, travel history, potential virus exposure scenario, contacts with known infections, and timelines of symptom onset, quarantine, infection confirmation, and hospitalization. These cases can be considered the baseline COVID-19 transmissibility under extreme mitigation measures, and therefore, a reference for comparative scientific investigation and public policymaking.

## Abstract (summary)

**Table:** 

Measurement(s)	Epidemiological Factors • exposure • Demographics • travel history • Quarantine • Symptom Onset • Symptom • Hospitalization • Viral Infection
Technology Type(s)	digital curation • manual coding
Sample Characteristic - Organism	Homo sapiens
Sample Characteristic - Location	China

Machine-accessible metadata file describing the reported data: 10.6084/m9.figshare.13567553

## Background & Summary

In the global fight against the COVID-19 pandemic, high-quality data are needed for clinical understanding, mathematical modeling, and policymaking considerations. However, official statistics reported in most countries are presented as aggregated data, e.g., newly confirmed cases by day and without critical details of individual cases, thus hindering their scientific values^1^. On the contrary, starting January 18, 2020, the Chinese health authorities, e.g., local health commissions, began to report detailed epidemiological information of confirmed COVID-19 infections publicly. We monitored the disclosure channels of 27 provincial and 264 urban health authorities, collected all the public reports, extracted any information about individual cases, and curated the dataset in the presentation, with the help of 15 research assistants.

Our data are updated regularly. As of November 20, 2020, 12667 cases are identified with detailed epidemiological information, accounting for 71.1% of a total of 17814 infections in China outside Hubei Province. The information contained in the dataset includes the case’s **demographic information**, e.g., gender, age, and occupation; **travel history**, e.g., trace and timeline of mobility; **exposure to known infections**, e.g., when, where, how, and to whom exposed; **timelines of case admission**, e.g., dates of quarantine, symptom onset, hospital admission, and infection confirmation; and **other information**, such as clinical symptoms, mentioned in the public disclosure.

The cases reported outside the epicenter provide unique and crucial value for the study of disease spreading. The COVID-19 epidemic in China outburst in Wuhan and spread outside of the city to Hubei Province and other parts of the country with the help of large-scale human mobility during the traditional New Year holiday. The Chinese government swiftly enforced a series of nationwide travel bans, which restricted mobility between cities, starting with Wuhan on January 23, then Hubei Province and the entire country a few days later^2^. Despite these extreme mitigation strategies, the disease still managed to spread in more than 260 cities in China^3^. The highly coordinated nationwide travel bans can be regarded as a rare emergence measure, under which disease can only spread locally. Therefore, the virus transmission paths and individual cases’ epidemiological timelines that can be further constructed from the curated data are a baseline for the further comparative study of COVID-19 spreading in different countries, populations, and under different mitigation policies.

## Methods

### Data sources

Most of the provincial and urban level health authorities in China began to report detailed information regarding COVID-19 cases admitted in their jurisdiction, starting from mid-January. The reports were published via official websites and social media platforms. We collect the reports from 27 provincial and 264 urban health authorities in China outside Hubei province, starting January 19, 2020. The list of disclosure channels of local health authorities is available in our dataset^4^.

### Information extracted

A typical example of a case report reads as follows.

Patient Yue, female, 42, resident in Building C, XX housing estate, Tianjia’an District, Huainan City, Anhui Province. She arrived in Huainan from Hefei on January 20. She had close contacts with confirmed cases Fu and Yue in Fengtai County from 23–30 January. Showed fever symptom on February 1, admitted to Chaoyang Hospital on February 4, tentatively diagnosed with COVID-19 on February 5, confirmed on February 6. The patient is currently quarantined and treated in the destined hospital with mild clinical symptoms.

Five categories of detailed information can be extracted from the above disclosure, i.e., demographic information, past mobility trace, exposure to the virus or potential contact with known infection(s), the timeline of symptom onset, hospitalization, infection confirmation, etc., and clinical symptoms.

Specifically, demographic information includes age, gender, occupation, and place of regular residency. The regular residence place may or may not be the patient’s hometown but can be their place of work or school. For example, the demographic information extracted from the sample case is female, 42 years of age, a missing occupation, and a regular resident in Tianjia’an District.

The end of January 2020 was the time of Chinese New Year, during which many people who work away from their hometowns would return home for family gatherings. Therefore, mobility traces are abundant in reported cases. A trace includes any known travel history of the patient, including the departure place, transit place, and destination, usually where the patient was admitted to a hospital. The date when the patient arrived at the destination is also logged. The sample case’s mobility trace includes the departure place Hefei City, destination Huainan City, and the arrival date January 20.

The local health authorities reported the potential exposure to the virus and contacts to known infections with emphasis. The disclosure of such information intends to warn the public for potential exposure to the infection, high-risk venues, or activities. Cases are very often reported to have multiple risky activities. Therefore, these activities form a time window of virus exposure. We also log the place, activity, and any person involved in each activity. The demographic and mobility information of any contact is searched in other reports. If there is a unique match, their case IDs and relationship are extracted and coded. In the sample case, the patient had an exposure window from January 23 to January 30, and the two known contacts were found in other records, with familial relationship to the patient, e.g., mother and son.

The quarantine (self or mandatory) dates, symptom onset dates, hospitalization dates, and confirmation dates are extracted. Epidemiological timelines vary between different ways that the cases were discovered. We group the discovery methods into three types.

1. If a case went for medical help by themselves, we classify it as a *self-discovery*. The epidemiological timeline of a self-discovery usually has four stages: self-quarantine (optional) – symptom onset – hospitalization – case confirmation – public disclosure. Note that the date of hospitalization is the date when the case went for medical help for the first time.
2. If the case was discovered during large scale screenings, we consider it as a *passive discovery*. The epidemiological timeline of a passive discovery is usually: case confirmation – public disclosure. The dates of symptom onset are sometimes retrospected.
3. If the case was discovered during a mandatory quarantine, we record it as a *quarantine discovery*. The epidemiological timeline of a quarantine discovery can be mandatory quarantine – symptom onset (optional) – case confirmation – public disclosure. When the symptom onset field is missing or later than case confirmation, the case is usually an asymptotic infection.

For example, the sample case is a self-discovery case, with no quarantine date, the symptom onset date February 1, the hospitalization date February 4, and the case confirmation date February 6.

The local health authorities reported the clinical symptoms of the cases in some cases. About 90 different symptoms or a level of severity were mentioned. We classify them into 16 categories. The sample case was reported with only a mild level of severity with no particular symptom.

### Coding procedure

Fifteen research assistants helped with the following five-step information extracting procedure.

1. *Workload division*. The dataset is equally divided into seven proportions. Each proportion is assigned to a group with two research assistants.
2. *Preliminary coding*. Each group first codes 30 random records in their assignments. The two members in the group discuss and reach a consensus on any dispute in their coding.
3. *Coding*. For each group, the two members independently code the data. Their codings are regularly reviewed by bulks of 200–300 records (the workload of one or two days).
4. *Consistency check*. Upon any dispute in the bulk encodings, a coder from another group is asked to solve the inconsistency. The solutions are fed back to the initial coders to solve the inconsistency.
5. *Coding unification*. Finally, the phrasing and formats of all data fields are reviewed. Any coding mistakes are corrected, and subtle differences in phrasings are unified.

## Data Records

The line-list data are available at the figshare repository^4^. They are also simultaneously updated in our GitHub repository^5^. The data consist of 28 fields in 6 groups. Data fields and their content formats are shown in Table 1. The field names are self-explanatory. When a data field is not reported, an “NA” is filled. Dates are coded in format mm/dd/yyyy. The possible values of some data fields are elaborated as follows.

- *Occupation*. There are 154 different occupations mentioned in the original reports. The original phrases in the public disclosures are retained.
- *Place_Departure*. If a case has no travel history, this field is labeled “local.”
- *Arrival_Date*. Arrival date is the last known travel date of the case. Local health commissions trace the travel history of cases with their own standards. Some arrival dates can be earlier than one month before the public disclosure date.
- *Place_and_Event*. Reported activities that could potentially expose the patient in a high-risk environment. Data contents include the place and/or event of the activities. The most probable event is selected when multiple events are present.
- *Venue*. The coded venue for potential exposure activities. If the public disclosure from local health authorities mentioned the place of potential exposure explicitly, they are categorized into “Social place,” “Family,” “Workplace,” “Public transportation,” “School,” and “Hospital.” Otherwise, if “Indoor,” “Outdoor,” and “Public place” were mentioned, we record the venues as is.
- *With_Whom*. Possible values include “Classmates”, “Colleagues”, “Confirmed cases”, “Suspected cases”, “Family members”, “Fellow passengers”, “Friends”, “Returnee from XX” “Returnee from other place”, “XX personnel”, and “non-local personnel”, where XX is the name of a place.
- *Contact_ID_Relationship*. Types of relationships include “Husband”, “Wife”, “Son”, “Daughter”, “Mother”, “Father”, “Sister”, “Brother”, “Uncle”, “Aunt”, “Nephew”, “Niece”, “Grandmother”, “Grandfather”, “Grandson”, “Granddaughter”, “Son-in-law”, “Father-in-law”, “Mother-in-Law”, “Daughter-in-Law”, “Relative”, “Girlfriend”, “Boyfriend”, “Neighbour”, “Colleague”, “Other contact”.
- *Method_Discovery*. Depending on the orders of epidemiological events in the individual cases’ timeline, cases are grouped into self-discovery, passive discovery, and quarantine discovery. For self-discovery cases, their symptom onset date, hospitalization date, and confirmation date are chronological; for passive discovery cases, their dates of confirmation are prior or the same to hospitalization date and quarantine date; for quarantine discovery cases, their quarantine date should be prior or the same to confirmation date.
- *Symptom*. The phrasings of symptoms are rather diverse in the original reports, with a total of about 90 different phrases. We further classify these phrases into 16 categories, including asymptomatic and the symptom, sign, and diagnoses related to the somatosensory, respiratory, digestive, nerve, and circulatory systems. Eventually, only 10 categories appear in the data. Note that for cases reported asymptomatic, they may or may not show symptoms eventually.
- *Original_Text_EN*. The English version of the original case disclosures is translated using Google translate services.

**Table 1:** Data fields and their content formats.

Category	Data field	Data format
Demographic information	ID	Province_City-SerialNumber
	Age	Number
	Gender	Male/Female
	Occupation	Text
	Place_Residency	Text (Province_City)
Mobility trace	Place_Departure	Text (Province_City/“local”)
	Place_Transit	Text (Province_City)
	Place_Destination	Text (Province_City)
	Arrival_Date	Date
Exposure to virus	Earliest_Possible_Date	Date
	Latest_Possible_Date	Date
	Place_and_Event	Text
	Venue	Text
	With_Whom	Text
	Contact_ID_Relationship	Text (ID(relationship)&...)
Epidemiological timelines	Place_Admission	Text (Province_City)
	Method_Discovery	“Self”/“Passive”/“Quarantine”
	Date_Quarantine	Date
	Place_Quarantine	“Quarantine camp”/“Home”/“Hospital”
	Date_Symptom_Onset	Date
	Date_Hospitalisation	Date
	Place_Hospitalisation	Text
	Date_Confirmation	Date
	Date_Disclose	Date
Clinical symptoms	Symptom	Text
	Symptom_Severity	“Stable”/“Mild”/“Severe”
Other information	Original_Text_CN	Text
	Original_Text_EN	Text

## Technical Validation

### Completeness of cases with detailed information

The Chinese health authorities report daily increases and accumulated numbers of COVID-19 infections. Best efforts were made to ensure that the number of cases collected each day can add to the reported cases. As of November 20, 2020, 12667 cases are recorded in our dataset, accounting for 71.1% of the 17814 total infections. The reasons for missing cases in each province/prefecture are summarized and reported in the dataset^4^.

### Statistical inspection of data fields

**Fig. 1:**
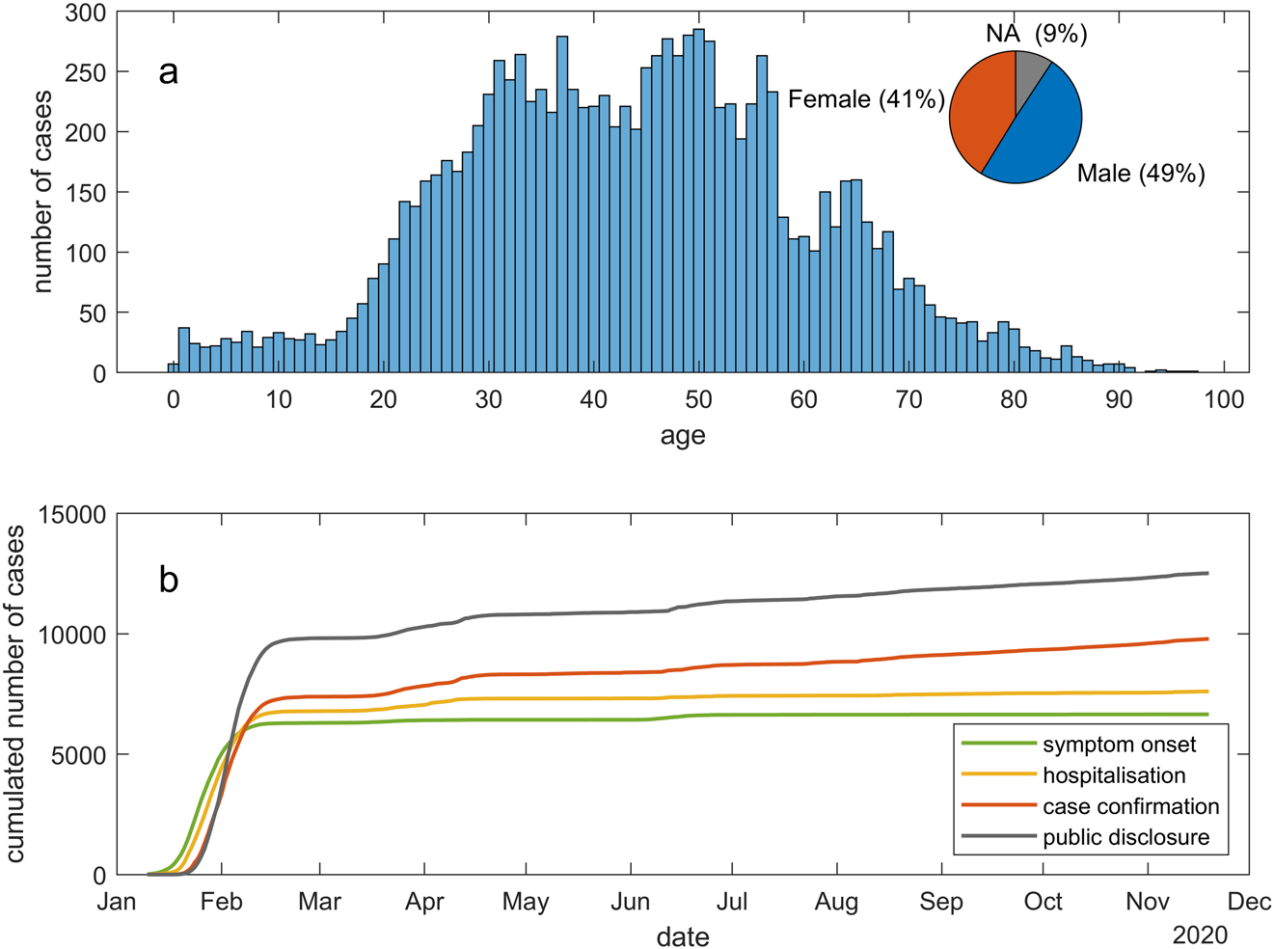
Statistical validation of several data fields. (**a**) The age and gender distributions of the cases. (**b**) The cumulative numbers of symptom onset, hospitalisation, infection confirmation, and public disclosure between January 10 and November 20.

**Fig. 2:**
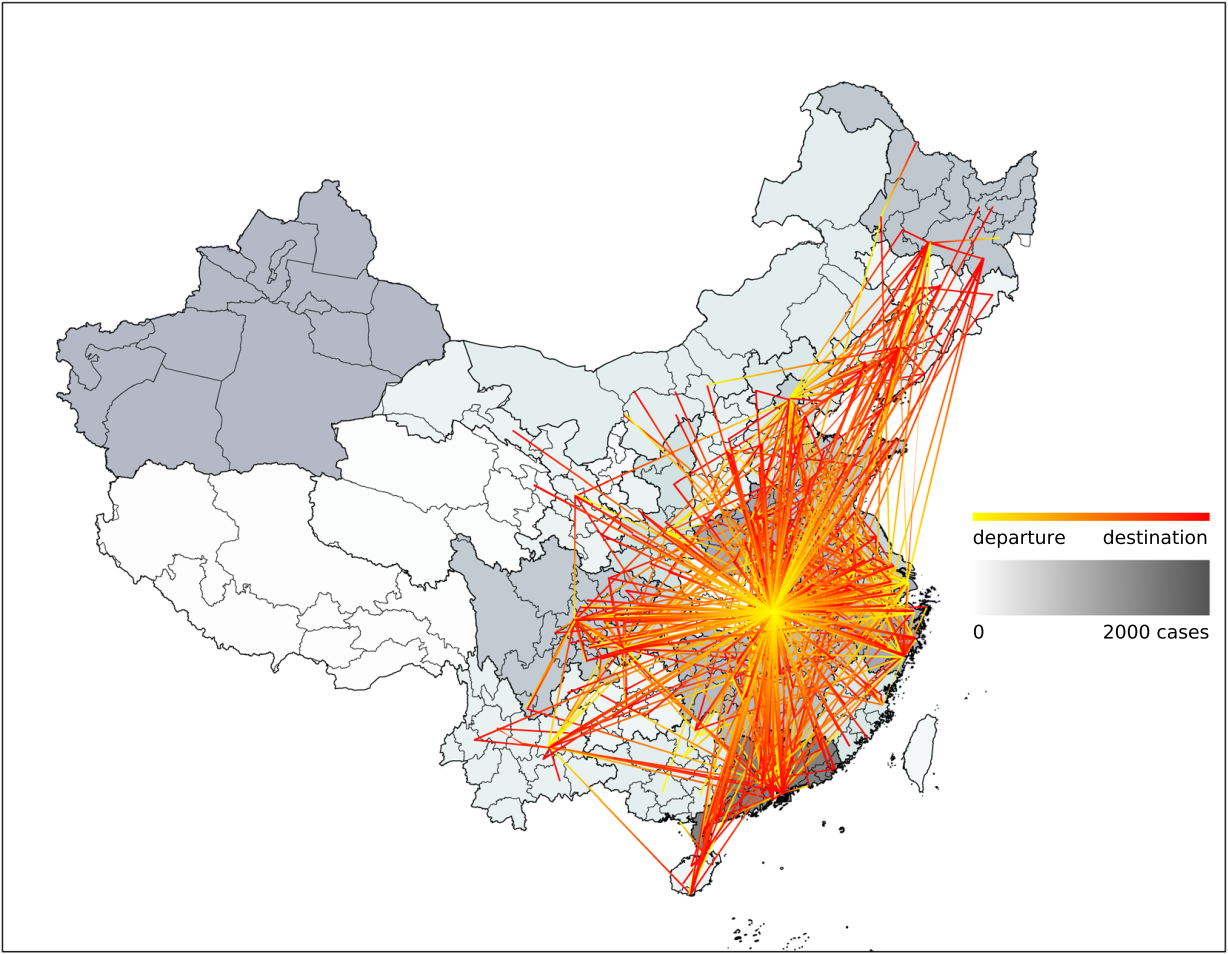
Mobility traces reported as of Nov 20, 2020. Lines with gradient color indicate the cases’ movements before arriving at the destination cities. Provinces in China are colored by their numbers of collected case reports. Note: only provinces and municipalities with reported cases are shown on the map; Hubei Province is not colored.

Here we plot the statistics of several essential data fields for visual inspection of data correctness. Figure 1(a) shows the age and gender distributions of the 12667 reported cases. The cases are concentrated in the mid-aged population, and that male infections are more than female infections. Figure 1(b) shows the numbers of symptom onset, hospitalization, infection confirmation, and public disclosure each day. Noticeable delays can be observed between symptom onset, hospitalization, and infection confirmation. The number of publicly reported cases surpassed that of confirmed cases at the end of January. Most of the cases reported but with no infection confirmation date were hospitalized with a suspected infection. Figure 2 shows the mobility traces of reported cases. Their departure, transit, and destination cities are linked with gradient color lines. Provinces are colored according to the numbers of cases collected in our dataset.

### Inter-coder reliability measures

Table 2 summarizes the number of possible values *C*, the percentage agreement between the two independent coders, Cohen’s kappa (*κ*)^6^, and the percentage of missing values for each of the data fields. The inconsistencies inside groups are solved before the dataset is published in the repository. Note that only the percentage of agreement is reported for the data fields with over 1,000 possible values.

**Table 2:** Inter-coder reliability measures as of Nov 20, 2020.

Category	Data field	*C*	Percentage agreement	Cohen’s kappa (*κ*)	Percentage missing
Demographic information	Age	97	98.1	0.98	13.0
	Gender	2	99.0	0.98	9.3
	Occupation	181	96.3	0.85	86.2
	Place_Residency	565	87.1	0.85	32.3
Mobility trace	Place_Departure	428	89.4	0.87	45.2
	Place_Transit	365	92.1	0.78	85.2
	Place_Destination	333	89.2	0.82	45.5
	Arrival_Date	309	92.7	0.87	50.4
Exposure to virus	Earliest_Possible_Date	115	94.8	0.83	89.0
	Latest_Possible_Date	175	96.1	0.95	49.8
	Place_and_Event	19	74.3	0.63	59.6
	Venue	9	89.9	0.74	77.2
	With_Whom	9	90.0	0.80	68.3
	Contact_ID_Relationship	>1000	94.4	—	76.2
Epidemiological timelines	Place_Admission	420	78.6	0.85	0.0
	Method_Discovery	3	91.6	0.90	30.5
	Date_Quarantine	287	92.9	0.83	64.5
	Place_Quarantine	3	93.8	0.90	56.5
	Date_Symptom_Onset	175	97.5	0.97	46.4
	Date_Hospitalisation	195	96.8	0.95	39.9
	Place_Hospitalisation	>1000	95.6	—	44.9
	Date_Confirmation	287	94.3	0.94	22.6
	Date_Disclose	304	95.1	0.95	1.0
Clinical symptoms	Symptom	16	96.8	—	62.5
	Symptom Severity	3	94.4	0.90	67.3

## Usage Notes

### Data integration

Two previously published datasets^7,8^ can be potentially integrated with our dataset. The dataset published along Zhang *et al*.^7^ recorded COVID-19 cases in China outside Hubei, starting from December 24, 2019, to February 9, 2020. Compared to this dataset, our dataset contains more detailed demographic information (with occupation and place of residence), travel history (departure, transit, destination cities, and arrival dates rather than yes/no), virus exposure (detailed scenarios and explicit contact persons rather than yes/no), and epidemiological timelines (with additional quarantine places and time, dates of case confirmation, and methods of case discovery). Our dataset also spans a longer time. The data fields in Xu *et al*.^8^ also intersect with our data fields, containing cases not only from China but also from the other parts of the world. However, since their data were sourced from health authorities’ brief reports, e.g., with only daily numbers and simple statistics such as gender, useful individual cases’ epidemiological information is sparse. For example, the missing rate of the date of symptom onset is 95.6% in their data compared to 46.4% in our dataset, as of November 20, 2020.

### Data update plan

The datasets will be updated on a bi-weekly basis. We plan to maintain and update the dataset whenever there is new confirmed local COVID-19 cases in China, until the pandemic ends. Meanwhile, we encourage the community to collaborate with us in this endeavor, by submitting data correction issues on the GitHub repository or join us in the coding group.

### Possible downstream tasks

The rich and complete individual case information in our dataset enables many possible downstream tasks that can be performed using our dataset. Apart from basic disease statistical properties such as the gender and age distributions of infections, the symptoms and the severity thereof, we identify three potentially valuable directions which require extra efforts in further manipulating the dataset, e.g., the reconstructions of epidemiological timelines, human-to-human transmission events and the spatial-temporal spreading trajectories of COVID-19.

#### Reconstruction of transmission events

The reconstruction of a transmission event requires the identification of an infector and an infectee. We have prepared close contact events between known infections as well as their relationships. However, the order of the case infections is not explicit and could only be inferred from their travel history and potential virus exposure scenarios. A possible way to identify an infector is that this case must have previous travel history or close contacts to any infections, e.g., our previous effort^9^. There are scenarios in which multiple cases have close contacts during the same period. The identification of transmission chains in a multiple-contact scenario is a long-recognized problem in epidemiology studies. We refer the readers to some of the previous studies, e.g., using statistical inference to determine the transmission chain^10^, or just using the first symptomatic infection as the infector for the whole cluster, e.g.^11,12^. The successful identification of transmission events would help the study of disease transmission risks in different scenarios and among people with different demographic features.

#### Reconstruction of epidemiological timelines

Epidemiological timelines, e.g., the delays between symptom onset, seeking health care and case reporting^13^, incubation period^14^, duration of virus shedding^15^, and serial interval^16^ are critical to understanding the temporal dynamics of COVID-19. Our data provide detailed records of the dates of possible exposure, symptom onset, quarantine, hospitalization, and confirmation of COVID-19 infections, which are the essential elements in the reconstruction of the epidemiological timelines. A small proportion of this dataset has been useful in discovering serial intervals of COVID-19^16^ and mitigation policies^17^. Another possible task is identifying asymptotic infections, who would have their dates of disease confirmation before symptoms onset or even without symptom onset. In this release of the dataset, we have also roughly classified the means of case discovery to self-discovery, passive discovery, and quarantine discovery, which can further help the reconstruction tasks.

#### Spatial-temporal spreading trajectories

Our dataset contains detailed travel history, including the places of departure, transit, and destination, as well as the arrival date. This information can be used to map the trajectories of COVID-19 spreading on the map. Using these trajectories with the whole Chinese population’s mobilization pattern during the 2020 Chinese New Year period^3^, one can further study the intensity of virus spreading between cities and precisely infer the proportion of the infected population in each geographical area, e.g., our previous effort^18^.

### Emergency monitoring with artificial intelligence

The authors would also like to take the opportunity and discuss the use of artificial intelligence in emergency response. Public disclosure from governments is becoming common and transparent in the world. Being able to extract information from the public announcements and integrate information from multiple sources is crucial to coordinated emergency response. The extraction of information from open texts, such as dates, places, names, and symptoms are typical named entity recognition task in natural language processing in artificial intelligence. In the effort of preparing this dataset, we have tried several established methods to identify desired targets from the text automatically. However, the precision is very low, partly due to the considerably different standards of reporting, phrasing, and degrees of disclosure, and therefore, we stick to manual coding for this dataset. On the one hand, we recognize that there is still a long way to go to apply artificial intelligence methods in not-well-defined tasks such as the present one. On the other hand, we hope that the original reports and the extracted information can be used as a training dataset and advance future natural language processing algorithms.

## Data Availability

No computer code was used in the data curation process.
